# Targeting the RANKL/RANK/OPG Axis for Cancer Therapy

**DOI:** 10.3389/fonc.2020.01283

**Published:** 2020-08-07

**Authors:** Jie Ming, Shane J. F. Cronin, Josef M. Penninger

**Affiliations:** ^1^Department of Breast and Thyroid Surgery, Wuhan Union Hospital, Huazhong University of Science and Technology, Wuhan, China; ^2^Institute of Molecular Biotechnology of the Austrian Academy of Science, Vienna Biocenter, Vienna, Austria; ^3^Department of Medical Genetics, Life Science Institute, University of British Columbia, Vancouver, BC, Canada

**Keywords:** osteoimmunology, RANKL/RANK/OPG, malignant tumor, targeted therapy, Denosumab

## Abstract

RANKL and RANK are expressed in different cell types and tissues throughout the body. They were originally described for their essential roles in bone remodeling and the immune system but have subsequently been shown to provide essential signals from regulating mammary gland homeostasis during pregnancy to modulating tumorigenesis. The success of RANKL/RANK research serves as a paragon for translational research from the laboratory to the bedside. The case in point has been the development of Denosumab, a RANKL-blocking monoclonal antibody which has already helped millions of patients suffering from post-menopausal osteoporosis and skeletal related events in cancer. Here we will provide an overview of the pathway from its origins to its clinical relevance in disease, with a special focus on emerging evidence demonstrating the therapeutic value of targeting the RANKL/RANK/OPG axis not only in breast cancer but also as an addition to the cancer immunotherapy arsenal.

## Introduction

In 1889, the “seed and soil” theory was first proposed by Stephen Paget for tumor metastases to distant organs (1). When tumor cells (“seeds”) leave their primary site of origin and spread, or metastasize, the microenvironment (“soil”) of the target organ is usually favorable for tumor cell anchoring and expansion of metastatic cells (2). Bone is not only the site for primary bone tumors such as giant cell tumors and osteosarcoma, but is also one of the most common distant metastatic sites for solid tumors such as multiple myeloma (MM), breast cancer, prostate cancer, and non-small cell lung cancer (NSCLC) (3), suggesting that the bone environment can serve as “soil” for tumor development and might also serve as a “seed” for further metastatic spread. Recent research on the bone microenvironment and its involvement in cancer biology has focused on the field of osteoimmunology, which includes the cross-talk between bone stromal cells (osteoblasts and osteoclasts) and immune cells. Identifying key players regulating bone homeostasis could pave the way for potential therapeutic cancer targets, in particular, to break the vicious circle of metastasis to the bones.

The receptor activator of the nuclear factor kappa-B ligand (RANKL, also known as TNFSF11), together with its receptor RANK (TNFRSF11A) (4), the decoy receptor osteoprotegerin (OPG; TNFRSF11B) (5), and the recently identified receptor Leucine-rich repeat-containing G-protein-coupled receptor 4 (LGR4) (6), has been shown to play critical bottleneck functions not only in regulating bone metabolism but also in immunity and tumorigenesis. In this review, we will briefly introduce the key functions of the RANKL/RANK/OPG axis in maintaining bone homeostasis and regulating immunity. Furthermore, we will discuss the role of this pathway from primary tumorigenesis to cancer metastasis with particular attention to breast cancer and the hormonal control of this pathway. We will also discuss recent data pointing to the RANKL/RANK axis as a novel therapeutic target in BRCA-mutated breast cancers and as a novel promising cancer immunotherapy agent.

## RANKL/RANK/OPG and Bone Homeostasis

**Figure 1:**
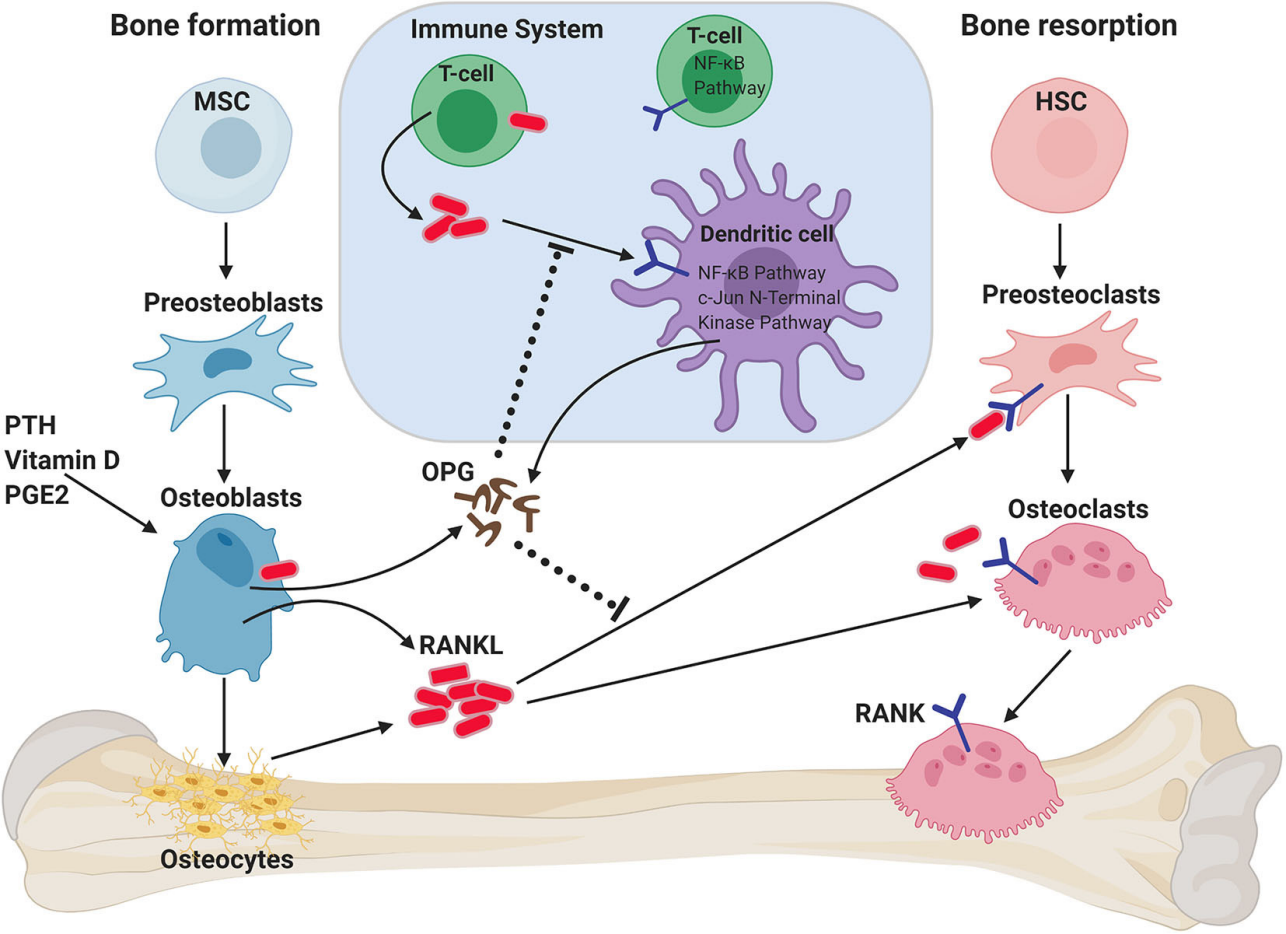
Role of RANKL/RANK/OPG axis on bone homeostasis and immune system. RANKL is secreted by osteoblasts and osteocytes when stimulated by parathyroid hormone (PTH), vitamin D, and/or prostaglandin 2 (PGE2). RANKL binds to RANK on the membrane of osteoclast progenitors (preosteoclasts), which results in bone resorption by mature osteoclasts. Osteoprotegerin (OPG) binds to RANKL, thus inhibiting RANK signaling and bone resorption. RANK/RANKL also plays a role in immune cell regulation and the crosstalk between both systems (termed osteoimmunology). T cells can also express RANKL, which can both act on preosteoclasts, but can also act on dendritic cells (DCs) to promote their survival and to prolong T–DC interactions. DCs can exhibit modulating effects on RANK-mediated osteoclastogenesis through the secretion of OPG. HSC, hematopoietic stem cell; MSC, mesenchymal stem cell.

Bone provides strength and structure, protects vital organs, stores minerals such as calcium, and is essential in the production of hematopoietic cells. Bone homeostasis is maintained by the balance between mainly two types of cells: osteoblasts (derived from mesenchymal cells) which build bone; and osteoclasts (derived from bone marrow hematopoietic precursor cells) which resorb bone (7) (Figure 1). Osteoblasts act as both mechanical sensors, together with osteocytes, and coordinators for the bone remodeling process, which is controlled by local growth factors and systemic factors, for example, calcitonin or sex hormones such as estrogen (8). The pathological imbalance between bone formation and resorption leads to the development of local or systemic bone diseases such as osteopetrosis and osteoporosis (9). The interaction and communication between osteoclasts and osteoblasts is intricately regulated in feedback loops to maintain bone homeostasis, and this constant remodeling process of the bone matrix is critical for healthy bone strength and efficient hematopoiesis (7, 10, 11).

RANK (TNFRSF11A, OFE, ODFR, TRANCE-R, ODAR, CD265) (4, 12, 13) and RANKL (TNFSF11, TRANCE, ODF, and OPGL) (14–17) are a receptor-ligand pair of the TNF receptor superfamily discovered at the end of the last millennium and were identified as key regulators of osteoclast development and bone metabolism (12, 18) (Figure 1). Factors that can induce bone resorption, such as the sex hormone progesterone, vitamin D3, PTHrP, IL-1, IL-11, IL-17, or TNF-α (19–23) act on osteoblasts to induce RANKL expression, which then binds to its receptor RANK on the surface of osteoclast progenitor cells, inducing pre-osteoclast differentiation into multinucleated, fully-functional osteoclasts. RANKL also plays an important role in the continued survival and function of osteoclasts (24–27) (Figure 1). RANKL is produced as a membrane-bound protein which can also be shed as a soluble trimeric protein (28). Sheddase-resistant RANKL mice have been generated, in which soluble RANKL is undetectable in the circulation (29); bone mass or bone structure was not affected during development in these mice, but adult mice displayed reduced osteoclast numbers and increased cancellous bone mass. Importantly, the bone loss caused by estrogen deficiency was unaffected by the lack of soluble RANKL. Thus, these data show that it is the membrane-bound form of RANKL which is largely responsible for the physiological functions of RANKL, although the soluble form can contribute to bone remodeling in adult mice (29).

Osteoprotegerin (OPG, TNFRSF11B) acts as a decoy receptor for RANKL and is induced by estrogen, IL-4 or transforming growth factor beta (TGF-β) (30). OPG competitively binds to RANKL, thereby interfering with RANKL–RANK interactions and blocking bone resorption (5, 31–35). The relative levels of OPG and RANKL are precisely controlled to ensure healthy bone. During pathological conditions such as menopause-related osteoporosis, decreased estrogen levels result in decreased OPG and subsequently increased RANKL, resulting in enhanced osteoclast activation and bone loss (36). Recently, leucine-rich repeat G protein-coupled receptor 4 (LGR4) was identified as an additional receptor for RANKL (6). Similar to RANK, LGR4 is expressed on osteoclasts, but unlike RANK, LGR4 is a negative regulator for osteoclast differentiation. Therefore, both OPG and LGR4 are endogenous inhibitors of RANKL/RANK signaling. A recent study has shown that RANKL reverse signaling from osteoclasts to osteoblasts couples bone resorption to bone formation processes (37). This is achieved through the secretion of small extracellular vesicles from osteoclasts that contain RANK. The authors showed that these RANK^+^ vesicles bind membrane-bound RANKL on the osteoblasts and thereby promote bone formation by triggering RANKL reverse signaling via activation of Runt-related transcription factor 2 (Runx2). Targeting RANKL reverse signaling represents a novel strategy to avoid the reduced bone production associated with inhibition of osteoclastogenesis (37).

As RANKL is an important regulator of bone loss in bone metastases (associated with cancers such as multiple myeloma) and in postmenopausal osteoporosis, a specific, fully human IgG2 monoclonal RANKL antibody (mAb) has been developed, which neutralizes the activity of RANKL, which has been designated as Denosumab. The efficacy of Denosumab has been confirmed in multiple clinical trials, and Denosumab therapy is now approved and widely used for the treatment of various bone-associated diseases (38–45).

## RANKL/RANK/OPG in the Immune System

Apart from bone homeostasis, the RANKL/RANK/OPG axis is also involved in various physiological immune processes. RANK was originally discovered on dendritic cells (DCs), and RANKL mediates the survival of DCs (46). The interaction between activated T cell-derived RANKL and RANK expressed on DCs increases the antigen-presenting capabilities of the latter, thus augmenting the number and cell cycle of antigen-specific T cells as well as enhancing the immune response of memory T cells (4).

Interestingly, phenotyping of *rankl* and *rank*-deficient mice revealed a complete absence of peripheral lymph nodes but intact spleen and Peyer's plaque structures (47–50). Subsequent studies have found that during embryogenesis, RANKL is expressed by hematopoietic lymphoid tissue inducing (LTi) cells and mesenchymal lymphoid tissue organizer (LTo) cells (51–53). RANKL has been demonstrated to stimulate lymphotoxin (LT) expression and regulate LTi cell accumulation. Furthermore, RANKL also triggers the proliferation of adult lymph node stroma, indicating that RANKL may directly activate LTo cells (51–54). In the thymus, the RANKL/RANK pathway is critical for CD80^+^ AIRE^+^ medullary thymic epithelial cell (mTEC) maturation involved in central immune tolerance (9, 49, 55). RANK-deficient mice display mild autoimmunity at an advanced age (4). RANKL/RANK activation in lymphatic endothelial cells (LECs) is important for the tissue-resident macrophages, namely, sinusoidal macrophage maturation not only during embryogenesis but also after inflammation-induced loss of these cells (56). Moreover, group 3 innate lymphoid cells (ILC3s) in the intestine use RANKL-RANK interactions to control their own abundance and intestinal homeostasis. Genetic ablation of RANKL specifically in IL3C cells leads to an increased number of these cells with enhanced levels of pro-inflammatory cytokines such as interleukin-17A (IL-17A) and IL-22 during intestinal infection (57).

Human patients carry *RANK* mutations and mice lacking RANKL or RANK exhibit a defect in B cell development, resulting in a significant reduction in B cell numbers (47, 49); however, these effects might be indirect because in the mouse, tissue-specific deletion of *Rank* in B cells showed no difference in function nor development of B cells, and blocking RANK/RANKL with Denosumab does not apparently affect B cell physiology in osteoporosis patients (58, 59). In addition, reports using B-cell-specific *rankl*-deficient mice have shown that B cell-derived RANKL increases osteoclast numbers and bone loss brought on by estrogen deficiency (60). Overexpression of RANKL in keratinocytes results in functional alterations of epidermal dendritic cells and systemic increases in regulatory CD4^+^CD25^+^ T cells (Tregs) numbers (61). Therefore, environmental stimuli can rewire the local and systemic immune systems via RANKL (61). The RANKL/RANK system is also involved in M (microfold)-cell development, a specific antigen-sampling cellular subtype found in the intestine, as mesenchymal cells produce RANKL that can directly interact with intestinal epithelial cells to regulate M cell differentiation (50, 62–64). Inhibition of mesenchymal RANKL impairs M cell-dependent antigen sampling and B cell-dendritic cell interaction in the subepithelial dome (SED), resulting in decreased IgA production and microbial diversity (63). In addition, B cells are absent in cryptopatches (CPs) and isolated lymphoid follicle (ILFs) formation was abrogated in *rankl* null mice (51).

Whether B cells or T cells are essential for bone loss is still controversial. Ovariectomy has been shown to enhance T cell-dependent TNF-alpha production in a bone loss mouse model because of the enhanced macrophage colony-stimulating factor (M-CSF) and RANKL (65, 66). In contrast, another study suggested T cells are not involved in ovariectomy-induced trabecular bone loss (67). Nevertheless, it has been reported in postmenopausal women that increased T cell activity and increased RANKL production by T cells are associated with osteoporosis (68, 69). Furthermore, studies in conditional knockout mice to specifically eliminate RANKL in B cells or T cells have shown that RANKL produced by B cells, but not T cells, leads to bone loss by the induction of osteoclastogenesis (60). The lack of mature B cells does not prevent bone loss (70), suggesting that RANKL is derived from immature B cells. Moreover, it has been reported that deletion of *rankl* in T cells does not change the number of T cells but results in impaired mature B cell numbers in the bone marrow, suggesting that RANKL might promote B cell maturation via paracrine signaling (60).

## RANKL/RANKL/OPG in Mammary Gland Physiology and Breast Cancer

Breast cancer is the most prevalent female malignancy (71). Studies based on large populations have shown that women who receive estrogen plus progesterone hormone replacement therapy (called combined HRT) are more vulnerable to breast cancer compared to women who receive estrogen only (72–74); furthermore, progesterone levels have been demonstrated to be an independent risk factor for increased breast cancer incidence (74, 75).

**Figure 2:**
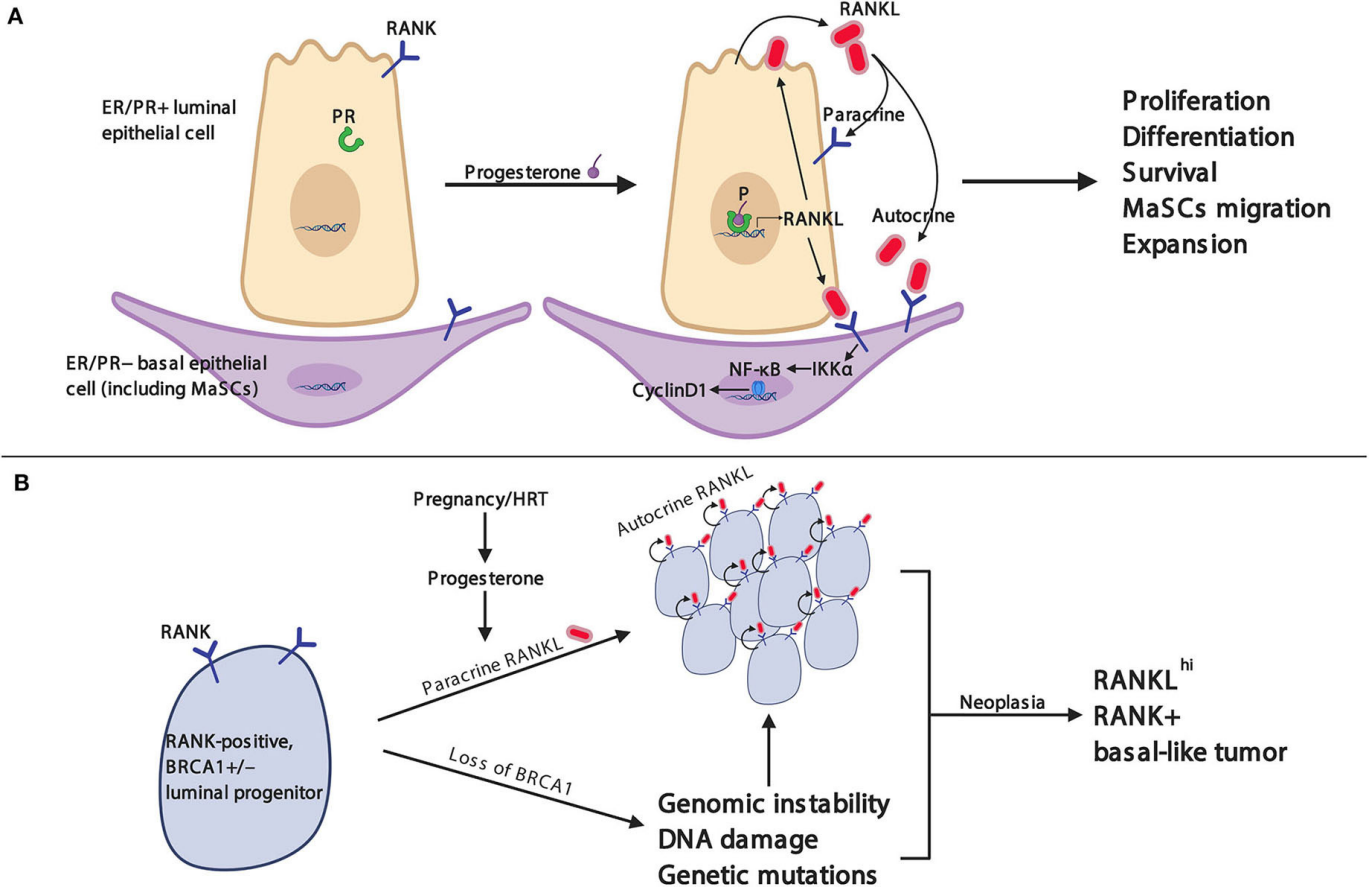
RANK/RANKL pathway in mammary gland physiology and breast cancer. **(A)** RANK is constitutively expressed on the membrane of luminal and basal epithelial cells including mammary stem cells (MaSCs). Stimulation with progesterone induces RANKL expression and secretion in progesterone receptor (PR)-positive luminal epithelial cells. RANKL binds in an autocrine fashion to RANK on luminal epithelial cells, which stimulates further RANKL expression, and in a paracrine fashion to RANK on basal epithelial cells, resulting in enhanced RANK expression on basal mammary epithelial cells and the activation of the IKKα-NFκB–cyclin D1 signaling axis to induce a variety of physiological responses necessary for mammary gland development. **(B)** Heterozygous *BRCA1* mutation-carrying women can spontaneously lose the remaining wild-type *BRCA1* gene from somatic mutation or epigenetic silencing. Subsequently, loss of BRCA1 protein can result in increased genomic instability, DNA damage, and genetic mutations (e.g., *TP53*). Progesterone, as well as synthetic progestins, up-regulate RANKL expression in PR^+^ luminal breast epithelial cells which stimulates RANK-mediated cell proliferation of adjacent progenitor cells as discussed in **(A)**. Altogether, the genotoxic stress and amplified proliferation cues culminate in uncontrolled proliferation and the development of breast cancer.

In *rankl* knockout mice, our group was the first to report that during pregnancy, RANKL deficiency results in a total block in the development of lobuloalveolar milk-secreting structures (76). Whereas, estrogen triggers the expansion of the mammary epithelium in puberty, progesterone drives the proliferation of mammary epithelial cells in the estrous cycle and, in pregnancy, induces the growth and differentiation of the mammary epithelium into ultimately milk-secreting acini (77) (Figure 2). Mechanistically, progesterone induces progesterone-receptor (PR)-positive mammary epithelial cells to express RANKL, resulting in the proliferation of neighboring RANK^+^ mammary epithelial progenitor cells in an autocrine and also paracrine fashion (78–80). Moreover, RANKL can induce the proliferation of RANK-positive ductal epithelial cells through the induction of R-spondin (80). Therefore, RANK and RANKL link sex hormones to mammary progenitor cell proliferation during the estrous cycle and in pregnancy (81) (Figure 2).

Clinically, an increase in serum progesterone and RANKL levels is associated with an increase in breast cancer risk in postmenopausal women (82). Higher concentrations of soluble RANKL are positively correlated with an increased risk of estrogen receptor-positive but not estrogen receptor-negative breast cancer, indicating that the RANK/RANKL/OPG axis may be involved in the tumorigenesis of ER^+^ breast cancer (83). Indeed, in a hormone-induced spontaneous mouse breast cancer model, RANKL is critical for the development of sex hormone-driven breast cancer (84). Deletion of RANK and Ikkα, a key downstream regulator of the RANK signaling pathway in mammary epithelial cells, also significantly delayed progestin (MPA) and DNA-mutation (DMBA)-induced mammary tumor formation, further indicating that the RANK/RANKL pathway drives breast cancer (85). Furthermore, the selective inhibition of RANKL by RANK-Fc not only attenuated breast tumor progression in a hormone- and carcinogen-driven mouse breast cancer model but also decreased the progression of breast cancer in a transgenic spontaneous tumor model (86).

*BRCA1* and *BRCA2* mutations are the most prevalent genetic drivers for hereditary breast cancer in humans. Interestingly, women with germline *BRCA1/2* mutations usually exhibit higher progesterone and estrogen levels during the gestational phase of the estrous cycle compared to women without these mutations (87). Inversely, decreased serum OPG levels are associated with increased breast cancer incidence (88). Moreover, high levels of RANK expression were observed in breast cancer samples from premalignant lesions and patients with *BRCA1* mutations (89, 90); SNP data analysis from the Cooperative Tumor Gene-Environmental Research (iCOGS), including approximately 15,200 *BRCA1* and 8,200 *BRCA2* mutation carriers, identified 6 SNPs which were significantly associated with breast cancer risk at the *TNFRSF11A* locus (encoding RANK) (90). Altogether, these human data strongly support the idea that the RANKL/RANK/OPG axis is intimately involved in the tumorigenesis of *BRCA1/2* mutation-driven breast cancer.

Subsequent animal studies provided direct evidence that RANKL and RANK are critically involved in the oncogenesis of *BRCA1* mutation-driven hereditary breast cancer (90) (Figure 2). Genetically engineered mice carrying *Brca1* and *Tp53* mutations showed hyperproliferation and malignancy in their mammary glands at 4 months of age; the inactivation of the RANKL/RANK pathway in these mice largely prevented the occurrence of malignant tumors and resulted in significantly prolonged survival. Additionally, the pharmacological blockade of RANKL using RANK-Fc completely abolished the development of precancerous lesions in the *Brca1/Tp53* double-mutated breast cancer model (90). Amplification of RANK-expressing mammary duct progenitor cells can be found in the non-tumor breast tissue of *BRCA1* mutant carriers, and these cells have similar molecular characteristics as basal-like breast cancer cells (90). RANKL inhibition also significantly suppressed the proliferation of tumor organoids derived from *BRCA1* mutant human breast biopsy specimens, and RANKL/RANK pathway blockade strongly reduced tumorigenesis in patient-derived xenograft (PDX) breast tumor mouse model (91). Thus, independent work among different laboratories, using different mouse models as well as studies using human breast epithelial progenitor assays, has led to the same conclusion: RANKL/RANK affect mammary progenitor cells and are critically involved in the *BRCA1*-mutation driven mammary tumorigenesis.

Therefore, we and others have proposed that the monoclonal antibody Denosumab (which specifically inhibits RANK/RANKL interactions) could potentially be used for the prophylactic treatment of breast cancer in *BRCA1/2* carriers (42). Indeed we posit that healthy women with *BRCA1* mutation will benefit, not excluding an effect on other TNBCs. In a pilot clinical study, termed BRCA-D, the proliferation marker Ki67 was significantly down-regulated in the breast biopsy of *BRCA1* mutation carriers who received short-term treatment with Denosumab, suggesting that RANKL inhibition may be a feasible method for the chemo-prevention of breast cancer in women with *BRCA1* mutations. This study requires additional patient data, which is currently ongoing (92). Another clinical study, D-BEYOND, which aimed to investigate whether neoadjuvant RANKL inhibition therapy can reduce tumor proliferation in premenopausal early breast cancer patients (93), found no significant change in Ki67-positive tumor cells in the breast cancer tissues treated with Denosumab, but the density of tumor-infiltrating lymphocytes (TILs) was increased in the stroma and tumor tissues upon Denosumab treatment (94).

In addition to the now experimentally well validated role of RANKL/RANK/OPG in the sex hormone and *BRCA1* mutation-driven mammary cancer tumorigenesis, it has also been reported that this pathway can induce epithelial-mesenchymal transition (EMT) in breast cancer cells, as well as in prostate and endometrial cancers (95–98), suggesting that RANKL/RANK supports tumorigenesis in various epithelial cancers. Moreover, our group has recently reported on the role of RANKL and RANK in lung cancer. We demonstrated that the inactivation of rank in lung epithelial cells disrupts mitochondrial bioenergetics and significantly reduces lung cancer development, both culminating in increased survival (99). This genetic modeling in the mouse supports findings in human clinical trials in which RANK inhibition with the monoclonal antibody Denosumab resulted in prolonged survival, especially in patients with non-small cell lung cancer (NSCLC) adenocarcinomas and squamous tumors. Notably, this Denosumab-dependent survival advantage occurred in lung cancer patients irrespective of visceral metastasis, hinting that the underlying effects of RANKL/RANK blockade, in addition to those targeting the bone, are involved (100). Epidemiological reports have also uncovered gender differences, particularly in lung cancer with respect to etiology, progression, and treatment response, believed to be due to sex-related hormonal factors (101–103), though the underlying molecular mechanisms are poorly understood. We have recently shown in our experimental lung cancer model that by ablating the sex hormones in female mice, we could effectively eliminate the survival advantages brought about by loss of *rank* in the lung tumors. Furthermore, synthetic progesterone MPA-dependent enhanced lung cancer initiation required RANK expression. Together, these data suggest that the sex hormone regulation of RANKL/RANK could also explain the gender differences seen in human lung cancer.

## RANK/RANKL as Regulators of Metastasis

Studies have now shown that the RANKL/RANK/OPG axis plays a role in the progression of malignant tumors by promoting tumor cell migration (104) stimulating tumor neovascularization and promoting distant metastasis of tumor cells (105).

Disseminated tumor cells are responsible for the early metastasis of tumors, which frequently can be detected in the bone marrow of patients with malignant tumors. This “micrometastases niche” forms a favorable microenvironment for the development of metastatic spread, protecting cancer cells from various anti-tumor treatments and modulating anti-cancer immune responses, thereby allowing the tumor cells to escape immune surveillance (106). The tumor microenvironment is a complex milieu composed of distinct factors such as cytokines, extracellular matrix components, and various cell types such as fibroblasts, endothelial cells, and immune cells, all of which participate in cancer development, progression, and metastasis (107). In bone tissue, the tumor microenvironment includes immune and tumor cells, as well as osteoblasts and osteoclasts, all of which participate in a “vicious cycle” that accelerates osteolysis and cancer cell proliferation through, in part, the RANK/RANKL/OPG axis (2, 108). For instance, cancer cells can increase the expression of RANKL in osteoclasts by secreting parathyroid hormone-related peptide (PTH-rP) (23, 109). Tumor cells can also directly express RANKL and secrete cytokines such as interleukin (IL)-1α, 6, 8, 11; TNF-α; macrophage colony-stimulating factor (M-CSF); or prostaglandin E2 (PGE2), all of which promote osteoclast differentiation and survival, resulting in local osteolysis which supports metastatic growth (110–117). Subsequently, growth factors released by the bone matrix such as insulin-like growth factors (IGFs), fibroblast growth factor (FGFs), platelet-derived growth factor (PDGF), or bone morphogenetic proteins (BMPs) promote cancer cell proliferation (118–127). In addition to cytotoxic drugs and endocrine disruptive drugs, therapies targeting the RANK/RANKL/OPG axis exhibit direct and/or indirect anti-tumor effects by blocking the vicious cycle between bone and cancer cells (89, 128–131).

In a murine model of melanoma metastasis, it was found that for malignant tumors with RANK expression, RANKL produced by osteoblasts and bone marrow stromal cells could act as a chemical attractant and promote the migration and metastasis of malignant tumors to these sites (132). Similar effects were also found in malignant tumors such as breast cancer (97, 133–135), prostate cancer (136–138), and lung cancer (100, 104, 139). The activation of phospholipase C (PLC), protein kinase C (PKC), ERK, and phosphatidylinositol-3-OH kinase (PI(3)K) pathways were involved in RANK-induced tumor cell migration (140–143). RANK engagement by RANKL induces trimerization of the RANK receptor which then stimulates the recruitment and activation of the adapter protein TRAF6 via TRAF6-binding sites in the C-terminus of RANK's cytoplasmic tail. TRAF6 in turn complexes with many other downstream adapters and kinases to activate the aforementioned pathways. Moreover, the RANKL/RANK pathway was also shown to promote the formation of new blood vessels and regulate the tumor microenvironment at the primary tumor site to promote the migration of tumor cells into the bloodstream and for metastasis to distant organs (144–147).

In breast cancer, RANKL is also produced by Foxp3-expressing Tregs and tumor-associated macrophages (TAMs) that can affect tumor growth, tumor cell dissemination, and metastasis (148). RANKL expression on tumor-infiltrating regulatory T cells may also be involved in cancer metastasis (148). TAMs are either M1 or M2 macrophages, with M1 being anti-tumor and M2 TAMs promoting tumorigenesis. Importantly, M2 macrophages express RANK and are attracted by RANKL produced by the tumor microenvironment. The RANKL/RANK pathway in M2 macrophages can regulate the production of chemokines and promote the proliferation of Treg lymphocytes, which supports the immunosuppressive milieu within the tumor microenvironment (149).

Recently, it has been reported that estrogen-related-receptor alpha (ERRα), an important factor of cancer cell invasiveness, promotes breast cancer cell dissemination from primary mammary tumors to the bone (150). Intriguingly, RANK has been shown to be a target for ERRα. Furthermore, the meta-expression analysis of breast cancer patients has uncovered a positive association between metastases and ERRα/RANK expression as well as a positive correlation between ERRα and *BRCA1* mutation carriers, revealing a novel pathway whereby ERRα in primary breast cancer could promote early dissemination of cancer cells to bone (150). Moreover, it was recently shown that RANKL serum levels are significantly increased in breast cancer patients who developed bone metastases (*p* = 0.01) and patients within the highest quartile of RANKL had a significantly increased risk of developing bone metastases compared to those in the lowest (HR 4.62, 95%CI 1.49–14.34, *p* = 0.03) (148). This study further suggests a role of RANKL in breast cancer metastasis (151).

## Targeting RANKL/RANK in Human Cancer

In light of the different roles of the RANKL/RANK pathway in bone metabolism and immune system functions, therapy targeting this axis may not only control primary tumor development such as in the case of breast cancer and reduce bone metastasis which has been demonstrated in clinical trials (152, 153) but also exert a direct anti-tumor effect via regulating local tumor-associated immune responses, as observed in studies using the monoclonal RANKL antibody inhibitor Denosumab (154, 155).

In randomized clinical trials, Denosumab has shown rapid effectiveness by directly impairing osteoclast activity and inducing osteoclast apoptosis (156). Moreover, Denosumab was significantly more effective in reducing urinary N-terminal peptides, a biochemical marker for bone turnover, and more effective in delaying skeletal-related events (SREs), such as pathologic fractures, spinal cord compression, and hypercalcemia, which greatly affect quality of life in patients with breast cancer and castration-resistant prostate cancer (CRPC) bone metastases. However, the effect of Denosumab to delay SREs in patients with NSCLC and multiple myeloma (MM) patients with bone metastases is comparable to bisphosphonate drugs (129–131). Moreover, the benefit of Denosumab and bisphosphonates is not only restricted to osteolytic cancers such as breast, myeloma, and NSCLC but also evident in osteoblastic cancers. Recently it was demonstrated in osteoblastic cancers, such as prostate cancer, that Denosumab or bisphosphonate can affect the osteoclast/osteoblast balance in the “vicious cycle” of bone destruction induced by metastasized cancer cells (157), which highlights the potential rationale in treating osteoblastic cancer patients with Denosumab or bisphosphonates.

In a randomized phase III clinical trial comparing Denosumab and bisphosphonate zoledronic acid (ZA) in patients with solid tumors (breast cancer, prostate cancer, multiple myeloma) and bone metastases, the results showed that Denosumab was similar to ZA in preventing or delaying the onset of primary SREs (39, 131, 157). However, in non-small-cell lung carcinoma (NSCLC) (*n* = 702) treatment with Denosumab showed a significant improvement in overall survival (100). In these patients, no statistically significant SRE delay was observed in Denosumab-treated patients, suggesting that this survival advantage may be independent of the bone system (131). The result of a randomized phase III trial of multiple myeloma (MM) patients (*n* = 1718) also demonstrated the effectivity of Denosumab to reduce the occurrence of primary SRE events; moreover, the use of Denosumab significantly improved progression-free survival (PFS) (39). Whether this survival benefit is due to the decrease in the incidence of bone metastasis or whether Denosumab has other anti-tumor effects requires further research.

In the randomized placebo-controlled phase III ABCSG-18 trial, which enrolled 3,425 postmenopausal female patients with early hormone receptor-positive breast cancer, the first clinical fracture of the Denosumab-treated group was compared with the placebo group, and a significant protection of bone breaks was demonstrated (hazard ratio [HR] 0·50 [95% CI 0·39–0·65], *p* < 0.0001) (152, 158). A median follow-up of 72 months showed a significant improvement in the disease-free survival (DFS) in the Denosumab-treated group (HR = 0.823, 95% CI 0.69–0.98, Cox *p* = 0.026). These data suggest that adjuvant Denosumab can significantly improve the DFS rate of HR+ postmenopausal breast cancer patients (159). However, in another randomized phase III clinical trial of breast cancer, D-CARE, recent reports have shown that adjuvant Denosumab does not reduce the risk of breast cancer recurrence or death in early-stage breast cancer patients receiving standard adjuvant therapy (153). These inconsistencies, which could be explained by different cohorts for patient stratifications (e.g., more advanced early cases of breast cancer were included in the D-CARE trials as compared to the ABCSG-18 study), need to be further evaluated with larger cohorts of patients and multiple-center analysis. Importantly, a recent follow-up study of the ABCSG-18 trial confirmed the that blocking RANKL in an adjuvant breast cancer therapy setting not only markedly reduces the risk of breaking bones but also significantly reduces the reoccurrence of the breast tumors (152, 153). It should be also noted that although there was no difference in bone-metastases-free-survival in the D-CARE trial, Denosumab treatment significantly reduced the time to bone metastasis at the site of first occurrence (152).

## Denosumab as A Novel Cancer Immunotherapy

The field of cancer immunotherapy has paved the way for a new paradigm to combat cancer, by coaxing the body's own immune system to seek out, specifically target, and destroy cancer cells. Among the various approaches, immune-checkpoint inhibitors that target CTLA-4 as well as the PD1–PDL1 interaction, enabling an enhanced T cell-dependent anti-tumor response, have revolutionized the treatment of advanced melanoma and other cancers. However, using anti-PD1 monotherapy or the combination of anti-PD1 and anti-CTLA4 is still limited to a minority of cancer patients, and a vast majority of patients do not derive clinical benefit due to primary resistance (160). Thus, novel combinations in immunotherapy targeting different cellular mechanisms are needed for these patients.

As discussed above, targeting the RANK-RANKL axis using the RANKL inhibitor Denosumab offers a potential new avenue as a preventative treatment for breast cancer in women with germline *BRCA1* mutations. Recent data have recently hinted at the exciting possibility of further repurposing Denosumab from an anti-resorptive agent in bone diseases to cancer immunotherapy in combination with checkpoint inhibition (155, 161). Although large trials have not yet been conducted to evaluate the efficacy of combinatorial therapy involving Denosumab with checkpoint inhibition, few case studies have given exciting insights into potential synergistic effects mainly due to the fact that Denosumab is given for the prevention of skeletal related events (SREs) in patients with multiple myeloma, as well as bone metastases from solid tumors. Two case reports of patients with metastatic melanoma treated with Denosumab and ipilimumab (anti-CTLA4 therapy) induced potent and near complete clinical responses above what was expected for ipilimumab alone, suggesting that RANKL inhibition can enhance the anti-tumor effects of checkpoint inhibition (162, 163). Furthermore, a retrospective analysis based on US electronic health record data evaluated patients with melanoma or NSCLC bone metastases who received Denosumab at the same time as immune checkpoint inhibition (155, 164). These data support the notion that RANKL inhibition may enhance the activity of immune checkpoint inhibition, leading to improved tumor control in patients. Moreover, in line with findings from patient case reports and retrospective analyses, research using cancer models in mice have also demonstrated targeting the RANK-RANKL axis in combination with anti-CTLA-4 checkpoint inhibition and showed significant synergistic effects in immune-mediated tumor rejection in multiple tumor types (161, 165, 166).

**Figure 3:**
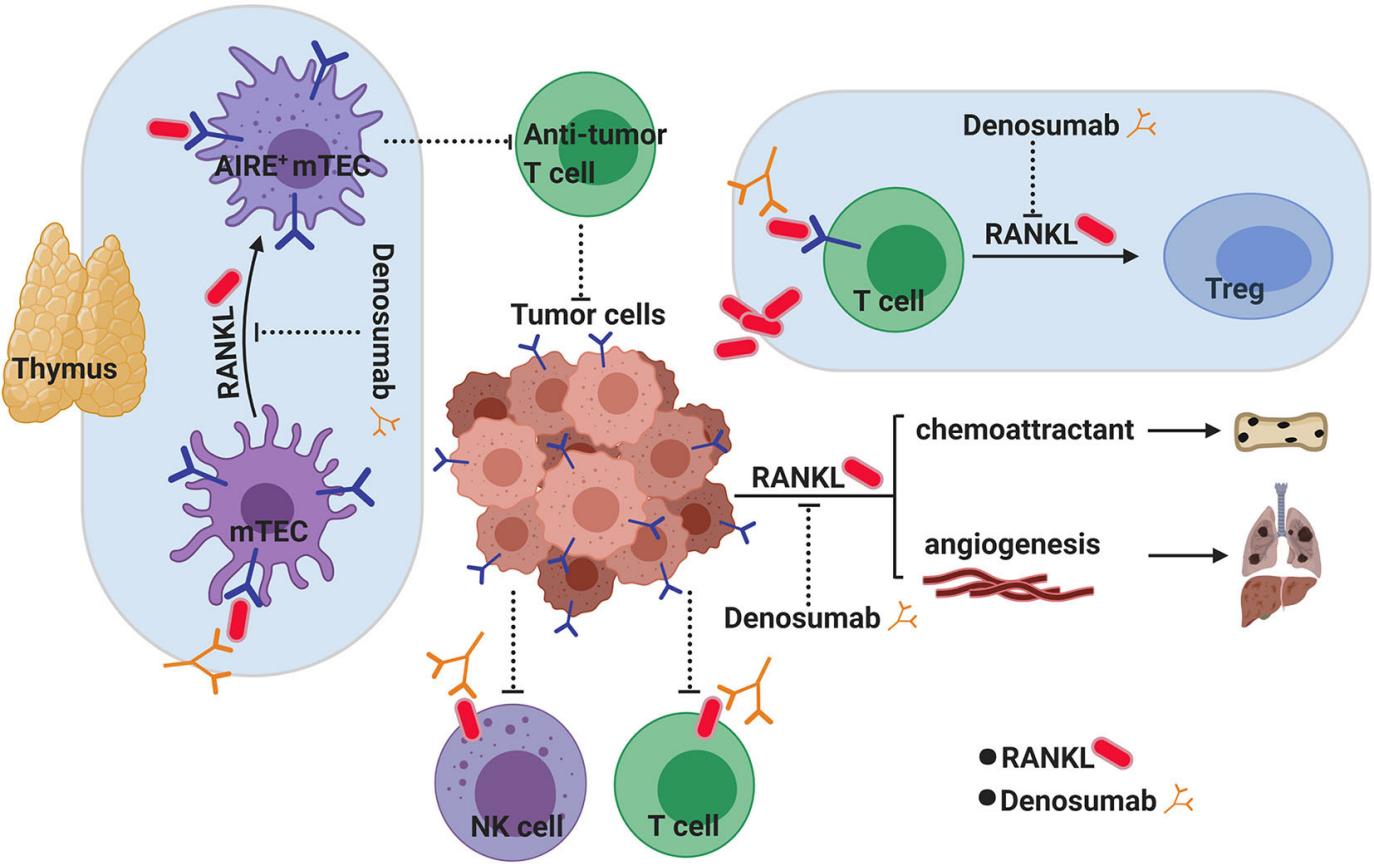
RANKL inhibition as a novel cancer immunotherapy. RANK/RANKL signaling has long been known to play an active role in supporting tumorigenesis through angiogenesis and metastasis both of which can be targeted through RANKL inhibition with Denosumab. However, blocking RANKL has recently gathered promise as a new avenue for cancer immunotherapy which may have complimentary and synergistic effects with known check point inhibitors in fighting cancer. How blocking RANKL achieves this is not known, although several hypotheses exist. In the thymus, the RANKL/RANK pathway is critical for CD80^+^ AIRE^+^ medullary thymic epithelial cell (mTEC) maturation and central tolerance. Temporarily blocking central tolerance through blocking RANK/RANKL by Denosumab could potentially increase the generation of more aggressive anti-tumor antigen T cells. Activated T cells and NK cells in the tumor microenvironment (TME) express RANKL on their surface, which can interact with RANK^+^ tumor cells to induce immunosuppression in these infiltrating cells. Blocking RANKL with Denosumab would overcome this suppression. Tumor-derived RANKL has also been suggested to play a role in converting RANK^+^ infiltrating T cells into immune-suppressing regulatory T cells (Tregs) in the TME.

How exactly Denosumab works as a cancer immunotherapy is not entirely clear (Figure 3). As detailed above, RANK is expressed in a variety of immune cells from macrophages to DCs, NKs, T cells, and myeloid-derived suppressor cells (MDSCs), and so by blocking the RANK–RANKL pathway, Denosumab may enhance the activity of these immune cells in the tumor microenvironment (TME) or indeed in the case of Tregs or MDSCs, reducing their immune-suppressive function. In terms of immune modulation, in patients receiving Denosumab for postmenopausal osteoporosis (PMO) a significant and prolonged (>6 months) increase in circulating B cells as well as transient increases in CD4+ T cells were observed (167). It has also been suggested that immunotherapy might modulate the expression or surface expression of RANKL. In a mouse model for colon cancer, anti-PD1 checkpoint inhibition resulted in increased surface expression of RANKL on tumor infiltrating lymphocytes (161). The anti-cancer activity of these T cells might be suppressed following engagement of RANKL on T cells and RANK expressed on other cells in the tumor microenvironment, and thus, Denosumab treatment would block this suppression.

Checkpoint inhibitors rely on modulating peripheral (i.e., extrathymic) immune tolerance to activate tumor-specific T cells after they have left the thymus. However, much less is known about how central (i.e., thymic) tolerance inhibits anti-tumor immunity. As mentioned above, the RANK–RANKL axis is required for the development of the AIRE-expressing medullary thymic epithelial cells (mTECs) of the thymus, involved in the “self” education of developing T cells (Figure 3). It has been shown that selectively targeting AIRE^+^ mTECs in the thymus thus transiently blocking central T cell tolerance can lead to the enhanced anti-tumor activity of T cells (168). As mTECs have a relatively short half-life of 2 weeks, the disruption of the RANK–RANKL axis with Denosumab could also temporarily block central tolerance mechanisms allowing the increased generation of anti-tumor T cells. This could potentially represent a novel mechanism for cancer immunotherapy to synergize with current treatment options of checkpoint inhibition. Whether Denosumab is affecting central tolerance or affecting cells in the local microenvironment of tumors is unclear at the moment, and further investigation is needed to delineate how RANK/RANKL signal inhibition contributes to enhanced anti-tumor immunity. However, given its well-known safety profile, anti-RANKL therapy makes an attractive candidate for repurposing as a more effective cancer immunotherapy.

## Conclusions

Although RANK and RANKL were first identified as critical players in bone remodeling, crucial roles of this pathway in controlling key aspects of the immune system and cancer as well as integrating sex hormone signaling to physiological adaptations have been uncovered and dissected in recent years. In the mammary gland, RANKL, induced by progesterone, signals to hormone receptor-negative RANK expressing epithelial cells to stimulate mammary progenitor cell expansion. Recently we and other groups have provided direct genetic and pharmacological proof that the RANKL/RANK pathway plays an essential role in the progression of familial *BRCA1*-mutated associated breast cancer. Since thousands of women have received the RANKL blocking antibody Denosumab to treat osteoporosis thus highlighting its safety profile, we believe that targeting the RANKL/RANK pathway with Denosumab is indeed a feasible strategy for the prevention of breast cancer in *BRCA1*-mutation carriers and may also be effective for other women at high risk for developing breast cancer. Importantly, a multinational phase III clinical trial using Denosumab to prevent the development of breast cancer in *BRCA1*-mutation carriers is currently being initiated. Recently, anecdotal evidence from case studies has raised the fascinating prospect of using RANKL inhibition as a novel cancer immunotherapy. Further research is however needed to reveal the underlying mechanisms of how RANKL blockage enhances anti-tumor responses of the immune system and whether combinatorial treatment with known checkpoint inhibitors can add to the arsenal of strategies in the fight against cancer.

## Author Contributions

JM, SC, and JP contributed to writing this review. All authors contributed to the article and approved the submitted version.

## Conflict of Interest

The authors declare that the research was conducted in the absence of any commercial or financial relationships that could be construed as a potential conflict of interest.
